# Migraine and tension type headache in adolescents at grammar school in Germany – burden of disease and health care utilization

**DOI:** 10.1186/s10194-015-0534-4

**Published:** 2015-06-04

**Authors:** Lucia Albers, Andreas Straube, Mirjam N Landgraf, Filipp Filippopulos, Florian Heinen, Rüdiger von Kries

**Affiliations:** Institute of Social Paediatrics and Adolescents Medicine, Division of Epidemiology, Ludwig-Maximilians-University Munich, 80337 Munich, Germany; Department of Neurology, Ludwig-Maximilians-University Munich, 81377 Munich, Germany; Department of Paediatric Neurology and Developmental Medicine, Hauner Children’s Hospital, Ludwig-Maximilians-University Munich, 80337 Munich, Germany

**Keywords:** Headache, Children, Adolescents, Health care, Burden of disease

## Abstract

**Background:**

Tension-type headache and migraine are among the most prevalent chronic disorders in children/adolescents. Data on health care utilization for headache in this age group, however, are sparse.

**Methods:**

In 1399 grammar school students (aged 12–19 years) with headache in the last six months in Germany a) the burden of disease for headache (mean intensity, mean frequency in the last three months and PedMIDAS means), b) medical care utilization defined by proportion of students consulting a physician in the last 12 months and/or taking analgetic drugs in the last three months by headache types (migraine and tension-type headache) and by burden of disease were assessed.

**Results:**

Primary headache substantially impaired daily living activities in adolescents which was mainly related to migraine. Medical care utilization and drug use, however, was low (consulting a physician: 12.0 %, 95 %-CI = [10.3-13.8]; taking analgetic drugs: 29.9 %, 95 %-CI = [27.5-32.4]) - even among students with severe headache (physician consultation: <35 %; taking analgetic drugs: <63 %). Two thirds of students with any headache and 40 % of those with migraine had neither seen a physician nor used analgetic drugs because of their headache in the preceding 12 months.

**Conclusions:**

Adolescents with headache might too rarely seek professional help for treatment of headache. Health promotion in adolescents should increase awareness for evidence-based treatment options for headache.

**Electronic supplementary material:**

The online version of this article (doi:10.1186/s10194-015-0534-4) contains supplementary material, which is available to authorized users.

## Background

Tension-type headache (TTH) and migraine are two of the most prevalent disorders in the world [1]. Migraine is the health disorder with the seventh highest impact on the quality of life and society [2]. Estimates on the prevalence and burden of disease for headache in children and adolescents vary considerably depending on age, setting, assessment etc. [3]. A recent review covering 64 headache and migraine studies in children and adolescents of the past 25 years reported an overall mean prevalence of 54.4 % for any headache and 9.1 % for migraine [4]. In the age group of 12 to 15 year old adolescents another review found a prevalence of 66 to 71% for at least one headache episode in the last three months, and 33 to 40 % for weekly headaches [5].

The burden of disease for headache in adolescents and children with respect to the impact on social functioning and quality of life is difficult to assess. A review on the impact of headache in children and adolescents stressed very inconsistent and poorly validated impact measures in most of the included studies [3]. Two important domains determining the impact of headache are suggested in this review: 1. Health status of the adolescents in terms of symptoms and illness control; 2. Functional status capturing the ability to perform activities that are essential to meet basic needs, fulfill roles and maintain well-being. In our study we assessed health status by headache frequency and pain intensity. The functional status was measured by the validated PedMIDAS questionnaire (Pediatric Migraine Disability Assessment Score) which assesses the impact of headache related to the quality of life in terms of the number of days in the last three months on which headache had a total or partial impact on schools or home life.

Optimal health care should allow coping with the challenges of headache with respect to participation in daily life and quality of life.

In adult populations medical care utilization in terms of consulting a physician and medication use is well evaluated [6–11]. For children and adolescents however, there are currently only very few data available which often date back at least one decade. There is only one study from Germany, which is more than nine years old [12]. From other countries there are two also older studies on health care utilization in adolescents in the United States [13,14]. There is only one recent study from Italy in younger children (aged 3 to 11 years) [15].

The aim of our study is to provide an updated assessment of a) the burden of disease for headache in grammar school students aged 12 to 19 years (both as an assessment of health status and functional status of the adolescents), b) medical care utilization of adolescents with headache in Germany defined by consulting a physician and/or taking analgetic drugs by headache types (migraine and TTH) and severity of disease.

## Methods

### Study population

1674 students of the 8^th^ to 10^th^ grade of 12 grammar schools in greater Munich (Germany) recruited for a headache intervention study (reported elsewhere [16]) filled in questionnaires about headache and risk factors for headache including questions concerning the burden of disease for headache and use of medical care and analgetic drugs. 1399 of these students, who reported at least one headache episode in the last six months, were included for this analysis.

### Assessment of headache, burden of disease, medical care and use of analgetic drugs

Headache was assessed by the question “Did you experience headache?”, which could be answered with “yes, in the last seven days”, “yes in the last three months”, “yes, in the last six months”, “yes, in the last 12 months” or “no”. Students, who reported headache in the last seven days, three or six months were assumed to suffer from headache. Students, who ticked “yes” were asked further questions assessing characteristics for classifying the headache as migraine and TTH (both confirmed and probable diagnosis) according to the International Classification of Headache Disorder-III beta (ICHD-III beta) [17]. A validated pain questionnaire for children and adolescents was used and specific questions were added to further classify headache subtypes as migraine or tension-type headache (TTH) according the classification of the International Headache Society [18,19]. Individuals with both probable migraine and probable TTH criteria were given a combined diagnosis of migraine plus TTH. All subjects with headache that did not match any of these criteria for primary headache were considered to have miscellaneous headache (MiscH).

The burden of disease for headache was assessed by the following two aspects: 1) Health status of the adolescents measured by the number of headache days in the last three months and the average pain intensity; pain intensity could be ticked on a Likert scale from 1= “very mild” to 10= “very severe”. 2) The functional status: disability due to headache was assessed using PedMIDAS, a standardized questionnaire consisting of the following six questions about how much headache is affecting day-to-day activity in the last three months [20]: (Q1), How many full days of school were missed in the last three months due to headaches?”, (Q2) “How many partial days of school were missed in the last three months due to headaches (do not include full days counted in the first question)?”, (Q3) “How many days in the last three months did you function at less than half of your ability in school because of headache (do not include days counted in the first two questions)?”, (Q4) “How many days were you not able to do things at home (i.e., chores, homework, etc.) due to headache?”, (Q5) “How many days did you not participate in other activities due to headaches (i.e., play, go out, sports, etc.)?”, (Q6) “How many days did you participate in these activities, but functioned at less than half your ability (do not include days counted in the 5th question)?”. The PedMIDAS total score sums up the number of days reported in each of the questions. Disability grading scores were determined by Hershey et al. based on the PedMIDAS total score [21] with total scores of 0–10 as “little to no disability”, 11–30 as “mild disability”, 31–50 as “moderate disability”, and total scores higher than 50 as “severe disability”. PedMIDAS was shown to be a sensitive, reliable and valid assessment instrument for headache related disability in children and adolescents [20].

Usage of analgetic drugs was assessed by the question “How often in the last three months have you taken analgetic drugs to treat your headache?” which could be answered with “never”, “rarely”, “sometimes”, “nearly every time” or “every time”. Use of analgetic drugs was assumed if students reported to take at least sometimes analgetic drugs. Furthermore students were asked if they have seen a physician because of their headache in the last 12 months, which could be answered with “yes” or “no”.

### Statistical analysis

For estimating the burden of disease for headache means with 95 % confidence intervals (95 %-CIs) were calculated for each of the PedMIDAS questions, the PedMIDAS total score, the pain intensity and the number of headache days in the last three months. To assess the headache type specific burden for migraine and TTH, means (with 95 %-CIs) were also given for students with a confirmed diagnosis of migraine and TTH (all other students including those with probable diagnosis of migraine or TTH were summarized as other headache types).

Group comparisons were made by comparing means or proportions and the respective binomial 95 %-CIs or formal chi-square test statistics. A sensitivity analysis was performed for different definitions of use of analgetic drugs: use of any analgetic drugs as compared to at least sometimes or regular. To assess if patients rather search sleep instead of taking drugs spearmen test and correlation coefficient between drug consumption and seeking sleep for pain relief was calculated.

## Results

Table 1 shows some demographic characteristics and the proportions of headache types in the study population: there was a slight female predominance; the mean age of the students was 14.5 years; the proportions of students in each grade were rather similar; regarding the headache types similar numbers of students with migraine and TTH were observed, however, confirmed diagnosis was more common for migraine than for TTH in our study sample. The mean time period since first onset of headache was about four years. In total only 17 students reported to have headache since less than one year.

**Table 1:** Demographic characteristics of the study population

Age (in years) –mean (SD)		14.51 (1.09)
Gender (female) - % (N)		56.94 (796)
Grade - % (N)	8^th^	32.59 (456)
	9^th^	38.96 (545)
	10^th^	28.45 (398)
Headache types	Confirmed migraine	18.73 (262)
	Probable migraine	10.86 (152)
	Confirmed TTH	9.86 (138)
	Probable TTH	19.16 (268)
	Migraine + TTH	23.09 (323)
	MiscH	18.30 (256)
Time period since the first headache attack (in years) – mean (SD)		4.18 (3.01)

Based on the disability grades of the PedMIDAS (Table 2) about 7 % of the students with any headache, 20 % with migraine and only 2 % with TTH were moderately to severely affected by their headache.

**Table 2:** Proportions of students in the PedMIDAS Disability Grades

	PedMIDAS Disability Grades			
	Little to none	Mild	Moderate	Severe
	(0–10)	(11–30)	(31–50)	(>50)
	% (N)			
Any headache	73.86	19.24	4.99	1.91
N = 1399	(1006)	(262)	(68)	(26)
Headache Types:				
Migraine	48.45	31.78	13.18	6.59
N = 262	(125)	(82)	(34)	(17)
TTH	83.33	14.49	2.17	0
N = 138	(115)	(20)	(3)	(0)
Other headache types N = 999	79.30	16.56	3.21	0.93
	(766)	(160)	(31)	(9)

The impact of headache on social activities assessed by the PedMIDAS questions is shown in Table 3: The number of total/partial absent days from school and of school days on which students were considerably restricted in paying attention in class because of their headache was on average 4.4 days in three months for students with any headache. Pain intensity was on average 5.5 points indicating a moderate pain intensity on average. The pain intensity differed between migraine and TTH patients (6.5/10 versus 4.9/10).

**Table 3:** Burden of disease in adolescents with headache

	PedMIDAS (given as number of days in the last three month)							Pain intensity (1 = “very mild” to 10 = “very severe”)	Number of headache days in the last three months
	Q1: absent days from school	Q2: partial absent days from school	Q3: school days with functioning < 50 % of their abilities	Q4: days where home activities could not be done	Q5: absent days from leisure activities	Q6: days with functioning < 50 % of their abilities in leisure activities	Total score (Q1 + Q2 + Q3 + Q4 + Q5 + Q6)		
	Mean [95 %-CI]								
Any headache N = 1399	0.57	0.90	3.17	1.81	1.64	1.76	9.33	5.33	8.56
	[0.50-0.65]	[0.77-1.03]	[2.85-3.49]	[1.61-2.01]	[1.49-1.79]	[1.56-1.96]	[8.61-10.05]	[5.30-5.37]	[8.35-8.78]
Headache Types:									
Migraine N = 262	1.08	1.82	6.67	4.33	3.46	3.50	19.19	6.51	13.06
	[0.88-1.29]	[1.35-2.29]	[5.46-7.89]	[3.49-5.16]	[2.93-4.00]	[2.70-4.30]	[16.45-21.93]	[6.44-6.57]	[12.38-13.73]
TTH N = 138	0.44	0.66	2.05	1.09	0.98	1.00	5.92	4.94	8.83
	[0.25-0.63]	[0.42-0.91]	[1.61-2.49]	[0.78-1.41]	[0.64-1.32]	[0.75-1.26]	[4.78-6.97]	[4.85-5.03]	[8.27-9.39]
Other headache types N = 999	0.46	0.69	2.43	1.27	1.26	1.41	7.18	5.08	7.34
	[0.37-0.54]	[0.56-0.82]	[2.14-2.73]	[1.11-1.43]	[1.12-1.39]	[1.23-1.59]	[6.56-7.80]	[5.04-5.12]	[7.11-7.46]

The average frequency of headache episodes was about nine days in the last three months for all students who had at least one headache episode in the last six months. A higher number of headache days was observed in migraine. Students suffering from migraine reported on average about 10 days with severe restrictions in school attendance in the last three months (absent days or >50 % restricted abilities to attend class). Health care utilization for headache assessed as seeing a physician in the last 12 months and use of analgetic drugs was low: 12.0 % (95 %-CI = [10.3-13.8]) of the students with headache have seen a physician and 29.9 % (95 %-CI = [27.5-32.4]) reported to take analgetic drugs. In migraine patients medical care use was higher: 24.4 % (95 %-CI = [19.3-30.1]) of migraine patients have seen a physician and 50.8 % (95 %-CI = [44.5-56.0]) took analgetic drugs.

Students who reported to take analgetic drugs never or only rarely, did not report to seek sleep more frequently instead – rather the opposite was observed: increasing use of analgetic drugs was positively associated with an incremental seeking of sleep for headache relief (r = 0.09, p = 0.0005).

Among the students with headache having seen a physician 49 % reported using analgetic drugs for management of acute headache episodes compared to 27 % of students not having consulted a physician (p < 0.001). 63.8 % of the students with any headache have neither seen a physician in the last 12 months nor took drugs. Among migraine patients 66.7 % of the students having consulted a physician reported to take analgetic drugs compared to 45.6 % of the students not having seen a physician (p < 0.005). 41.0 % of the migraine patients neither have seen a physician nor took analgetic drugs.

Irrespective of how the burden of disease was classified (PedMIDAS total scale >30, pain intensity >5 or frequency of more than 6 days of headache in the last three months), use of health care appeared to be low in general. Even in the more severe categories health care use did not exceed 60 % (physician consultation: 19 to 35 %; use of analgetic drugs: 19 to 28 %) (Table 4). As shown in a sensitivity analysis (Additional file 1: Table S1) defining use of analgetic drugs as “any use of analgetic drugs” instead of counting rare use of analgetic drugs as no use shifted these proportions about 20 % higher irrespective of the burden of disease due to headache observed.

**Table 4:** Frequency of medical care utilization by burden of disease for headache

		Having seen a physician because of the headache in the last 12 months (yes)	Use of analgetic drugs (sometimes or every time)
Burden of disease		%	
		(N)	
		[95 %-CI]	
PedMIDAS	moderate/severe (N = 93) (total scale >30)	35.48	62.77
		(33)	(59)
		[25.83-46.09]	[52.18-72.52]
	little to none/mild (N = 1259) (total scale ≤30)	10.25	27.61
		(129)	(349)
		[08.63-12.05]	[25.16-30.17]
Pain intensity (on Likert scale from 1 to 10)	>5 (N = 625)	20.00	43.06
		(125)	(270)
		[16.93-23.35]	[39.15-47.04]
	≤5 (N = 740)	4.73	19.05
		(35)	(141)
		[3.32-6.52]	[16.28-22.07]
Frequency of headache episodes	More than six headache days in the last three month (N = 529)	18.71	40.23
		(99)	(214)
		[15.48-22.30]	[36.03-44.53]
	At most 6 headache days in the last three months (N = 858)	7.81	23.48
		(67)	(201)
		[6.10-9.81]	[20.68-26.47]

The PedMIDAS scores for students who consulted and students who did not consult a physician in the last year is shown in Additional file 2: Figure S1 of the supplemental material: Students who consulted a physician because of their headaches were more likely to have “mild”, “moderate” or “severe” than “mild to none” PedMIDAS scores compared to students who did not consult a physician.

## Discussion

The impact of headache, especially migraine in adolescents was substantial accounting for considerable inability to attend school. However, medical care utilization was low - even among student with severe headache. Two thirds of students with any headache and 40 % of those with migraine had neither seen a physician in the preceding 12 months nor used analgetic drugs in the preceding three months.

The high burden of disease in children and adolescents with headache is in line with two previous reviews [3,4]. Three studies cited in these reviews assessed the burden of migraine by PedMIDAS and reported total scores of 17.8 to 44 days where children/adolescents were totally or partially disabled at home or at school because of migraine [20–22]. However, these studies were conducted in a clinical sample which could explain the higher number of days of impairment in two of these studies. Two other population based studies reported that students missed on average 7.2 school days in the last six months due to migraine, which is comparable to our study (about 3 days in the last 3 months) [23,24].

For children and adolescents data for headache health care utilization are sparse. Most studies date back to at least one decade. There is only one recent study from Italy on medication use in pre pubertal children [15]. In a 9 year old German study (12) use of analgetic drugs and physician consultation were compared in several disorders (stomach-, back-, orofacial pain) including headache (analgetic drugs use <25 %, physician consultation ≤17 %). No differentiation by type of headache is reported in this study.

A strength of our study and a new aspect is the analysis of actual data for health care utilization of adolescents with headache in Germany in relation to type and severity of headache. Furthermore, since the study was done in an area with general high availability of health care the low consultation rate seems to reflect the students’ unawareness of the benefit of medical consultation regarding their headaches rather than lack of treatment facilities. Headache type specific estimates were confined to categories previously shown to be stable such as confirmed migraine and TTH [25]. A further strength of our study is that the burden of disease for headache was assessed by two domains: The disability due to headache using an age-adapted, validated and reliable instrument (PedMIDAS), and additional the functioning level by considering pain intensity and frequency of headache of the adolescents [20]. This is important since the retrospective assessment by PedMIDAS may be fraught with bias in both directions: 1. Underestimation for non-school days due to headache on weekend days was shown [26]. 2. Due to the retrospective assessment of the PedMIDAS, recall bias might be possible; it has been shown, that the recall of the disability due to headache in the last three months overestimates the exact number of days compared to a headache diary [27]. A further limitation could be the failure to assess distress due to headache and quality of life directly by including a general quality of life measure (like i.e. KINDL) in our questionnaire [28]. However, in a former study in grammar school students in Munich strong restriction of the quality of life by headache, especially migraine has been shown [29]. Another measure for the burden of headache could have been the duration of the headache episodes, which unfortunately was only assessed by categories precluding calculation of mean values for simple assessment of the burden for any headache/headache types. Another weakness of the study pertains to the failure to ask for prophylactic therapy in our questionnaire and the specific analgetic drugs which were used to treat the acute headache attacks. However, prophylactic medications have to be prescribed by a physician and the number of patients consulting a physician was rather low. Thus missed prophylactic drug treatment is unlikely to be high in our sample.

Ascertainment of physicians’ visits in the last year only might be an underestimate. Questions about lifetime consultations because of headache as well as about the course of headache (unchanged, improved, deteriorated) during the previous year as compared to the year before may have been useful, but unfortunately was not assessed in our study. Furthermore emergence of headache might have been too recent to allow for a doctor’s visit. As only 17 students reported to have headache since less than one year, however, it seems very unlikely that students did not consult a doctor in the last year because the time since onset of headache was too short for making an appointment.

A further source of bias could be problems in understanding the difference between analgetic drugs and prescription medication in the students. However, analgetic drug in German means any drug used for pain relief irrespective of whether the drug is prescribed or OTC and therefore this seems rather unlikely.

Limited external validity is a weakness of this paper since the study was performed in grammar school students in an urban environment. However, since grammar school students usually have a higher socioeconomic family background, it is likely that access to health care in less privileged children with headache is even lower.

## Conclusion

Adolescents in Germany with primary headaches, specifically migraine, have a high burden of disease. Although health care utilization is higher in migraineurs (particularly among those more severely affected) than for other types of headache, almost 40 % of the students with migraine had neither seen a physician during the previous 12 months nor reported medical treatment. Health care promotion in adolescents should include elements to increase awareness for treatment options for headache.

## Additional files

Additional file 1: Table S1. Sensitivity analysis to assess possible differences by definition of use of analgetic drugs (any versus sometimes/every time). Additional file 2: Figure S1. Proportions of PedMIDAS scores in students who did and who did not consult a physician because of their headaches.

## Abbreviations

TTH: Tension-type headache
CI: Confidence interval
ICHD: International classification of headache disorder
