# Supplementary material for: Migraine and tension type headache in adolescents at grammar school in Germany – burden of disease and health care utilization
Source: J Headache Pain. 2015 Jun 4;16:52. doi: 10.1186/s10194-015-0534-4 (PMC4467810; doi:10.1186/s10194-015-0534-4)
Supplement: Additional file 1: Table S1. — Sensitivity analysis to assess possible differences by definition of use of analgetic drugs (any versus sometimes/every time). [file 10194_2015_534_MOESM1_ESM.doc]

Additional file 2. Table S1: Sensitivity analysis to assess possible differences by definition of use of analgetic drugs (any versus sometimes/every time)

|  |  | Use of analgetic drugs (sometimes or every time) | Use of analgetic drugs (any) |
| --- | --- | --- | --- |
| Burden of disease |  | % (N) [95 %-CI] | |
| PedMIDAS | moderate/severe (N = 93) (total scale >30) | 62.77 | 78.72 |
|  |  | (59) | (74) |
|  |  | [52.18-72.52] | [69.07-86.49] |
|  | little to none/mild (N = 1259) (total scale ≤30) | 27.61 | 50.24 |
|  |  | (349) | (635) |
|  |  | [25.16-30.17] | [47.44-53.03] |
| Pain intensity (on Likert scale from 1 to 10) | >5 (N = 625) | 43.06 | 64.27 |
|  |  | (270) | (403) |
|  |  | [39.15-47.04] | [60.38-68.03] |
|  | ≤5 (N = 740) | 19.05 | 41.49 |
|  |  | (141) | (307) |
|  |  | [16.28-22.07] | [37.91-45.13] |
| Frequency of headache episodes | More than six headache days in the last three month (N = 529) | 40.23 | 64.66 |
|  |  | (214) | (344) |
|  |  | [36.03-44.53] | [60.43-68.73] |
|  | At most 6 headache days in the last three months (N = 858) | 23.48 | 44.16 |
|  |  | (201) | (378) |
|  |  | [20.68-26.47] | [40.80-47.56] |
