# Supplementary figures and images for: Migraine and tension type headache in adolescents at grammar school in Germany – burden of disease and health care utilization
Source: J Headache Pain. 2015 Jun 4;16:52. doi: 10.1186/s10194-015-0534-4 (PMC4467810; doi:10.1186/s10194-015-0534-4)

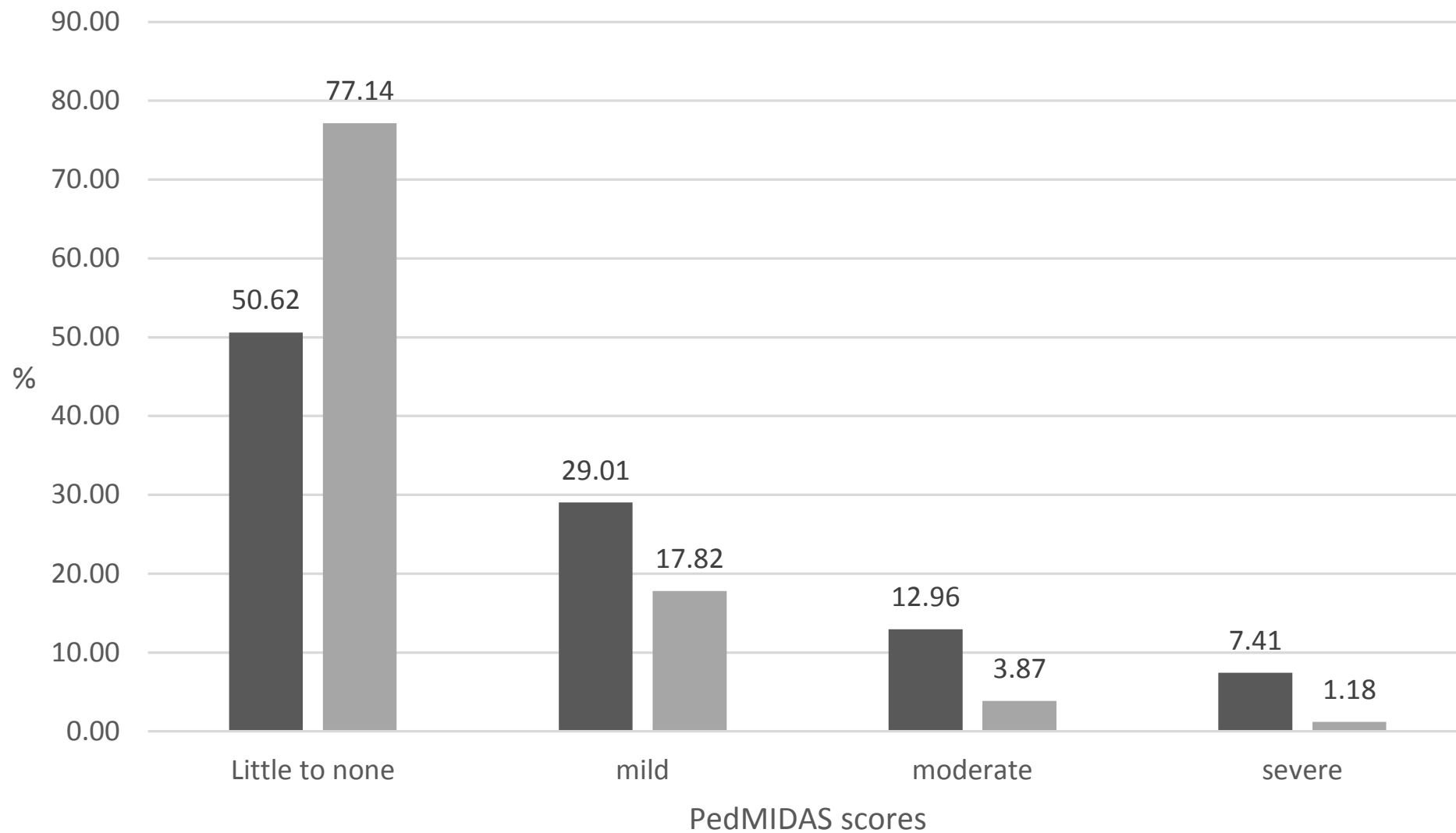

■ Students, who consulted a physician (N=162)

■ Students, who did not consult a physician (N=1190)

Supplement: Additional file 2: Figure S1. — Proportions of PedMIDAS scores in students who did and who did not consult a physician because of their headaches. [file 10194_2015_534_MOESM2_ESM.pdf]
